# Electroacupuncture enhances rehabilitation through miR-181b targeting PirB after ischemic stroke

**DOI:** 10.1038/srep38997

**Published:** 2016-12-14

**Authors:** Bin Deng, Fuhai Bai, Heng Zhou, Dandan Zhou, Zhi Ma, Lize Xiong, Qiang Wang

**Affiliations:** 1 Department of Anesthesiology, The First Affiliated Hospital of Xi’an Jiaotong University, Xi’an 710061, China; 2 State Key Laboratory of Military Stomatology & National Clinical Research Center for Oral Diseases & Shaanxi Key Laboratory of Oral Diseases, Department of Anesthesiology, School of Stomatology, Fourth Military Medical University, Xi’an 710032, China; 3 Department of Anesthesiology, Xijing Hospital, Fourth Military Medical University, Xi’an 710032, China

## Abstract

Recent studies have demonstrated microRNAs (miRNAs) and proteins are beneficial to axon regeneration, which may be involved in Electroacupuncture (EA) therapy against stroke. In this study, we aimed to determine the pivotal role of PirB in EA-produced rehabilitation against ischemic stroke; and to screen and investigate the potential miRNAs directly regulating PirB expression. The results showed EA treatment enhanced axon regeneration and new projections from the corticospinal tract at 28 d after cerebral ischemic reperfusion injury of rats. Then, we found EA decreased *pirb* mRNA and PirB protein expression in the penumbra within 28 days after reperfusion. The reduction of PirB expression facilitated neurite outgrowth after oxygen-glucose deprivation injury. The miRNA microarray showed the level of twenty kinds of miRNAs changed in the penumbra after EA administration. The bioinformatics study and luciferase assay verified miR-181b directly regulated *pirb* mRNA expression. EA increased miR-181b levels in the penumbras, and improved neurobehavioral function rehabilitation through miR-181b direct targeting of *pirb* mRNA to regulate the expression of PirB, RhoA and GAP43. In conclusion, we provide the first evidence that EA enhances rehabilitation against stroke by regulating epigenetic changes to directly act on its targets, such as the miR-181b/PirB/RhoA/GAP43 axis, which is a novel mechanism of EA therapy.

Stroke is the leading cause of adult disability in the United States and Europe^1^,^2^. Stroke lesions often result in permanent neurological deficits, which are caused by the destruction of a relatively broad region of the cortex^3^ and are accompanied by sensorimotor cortex damage in humans and experimental animals^4^,^5^. However, no single recovery aid is available for the rehabilitation of patients after stroke^6^.

Acupuncture has been used for stroke recovery in East Asia for centuries. However, scientific studies regarding acupuncture have only recently started to merge with Western scientific methods. Electroacupuncture (EA), or engrafted electric stimulation, is accepted as a common complementary therapy for stroke and post-stroke rehabilitation^7^. Although the efficacy of EA in stroke treatment remains controversial, most available evidence suggests that acupuncture promotes the recovery of neurological function and thus improves quality of life after stroke^8^,^9^,^10^,^11^. However, the underlying mechanism of the enhancement of neurofunctional recovery by EA after ischemic stroke remains to be elucidated.

In the days to weeks after ischemic injury, neurofunctional recovery has been associated with neural plasticity, including axonal sprouting and rewiring, the formation of new projections from the corticospinal tract (CST)^12^,^13^. Paired immunoglobulin-like receptor B (PirB) is a recently identified receptor of the following three major myelin inhibitors: Nogo-A, myelin-associated glycoprotein (MAG), and oligodendrocyte-myelin glycoprotein (OMgp) of axon regeneration^14^. PirB has been implicated in mediating the inhibition of neurite outgrowth after stroke and spinal cord injury^15^,^16^,^17^. Moreover, its inhibitory effect on axon regeneration is likely more important than that of Nogo-66 receptor (NgR1)^18^. Intriguingly, our previous study has demonstrated that PirB expression is up-regulated in the ischemic penumbra following transient focal cerebral ischemia in mice, which suggests that its expression in neurons plays an important pathological role in the inhibition of axon regeneration after stroke^19^. Interestingly, several studies have reported that the protective effects of EA on cerebral ischemic injury in rats might be closely associated with the down-regulation of Nogo-A and its receptor, NgR1, in the central nervous system (CNS)^20^,^21^,^22^. However, whether EA enhances neurobehavioral functional recovery via regulating PirB expression remains to be explored.

Most importantly, recent studies have revealed that a group of microRNAs (miRNAs) are involved in the mechanism employed by EA in the regulation of its target molecules after stoke^23^,^24^. The miRNAs are a class of endogenous, short (18 to 25 nucleotides) non-coding RNAs that bind to the 3′ untranslated regions (UTRs) of target mRNAs by complementary base pairing and prevent translation or destabilize the mRNAs to mediate their degradation or inhibit their translation^25^,^26^. The miRNAs can be activated in response to neuronal activity, and therefore, they offer a highly effective means of and play crucial roles in controlling the expression of proteins involved in both the developing and mature brain, specifically during neuronal differentiation^27^,^28^, axon regeneration^29^ and synaptic plasticity^30^. Thus, among the miRNAs with high levels of expression in the brain, those that regulate axon growth and target *pirb* mRNA in response to EA treatment for ischemic stroke need to be further explored.

To address these questions, the present study aimed to determine the pivotal role of PirB in EA-produced rehabilitation against ischemic stroke, and to screen and investigate the potential miRNAs, which can directly regulate PirB expression *in vivo* and *in vitro* study. This study is the first to reveal that EA exerts therapeutic effects through inducing epigenetic changes to regulate its targets, such as the miR-181b/PirB/RhoA/GAP43 axis.

## Results

### EA enhanced axon regeneration and CST projection after stroke

**Figure 1:**
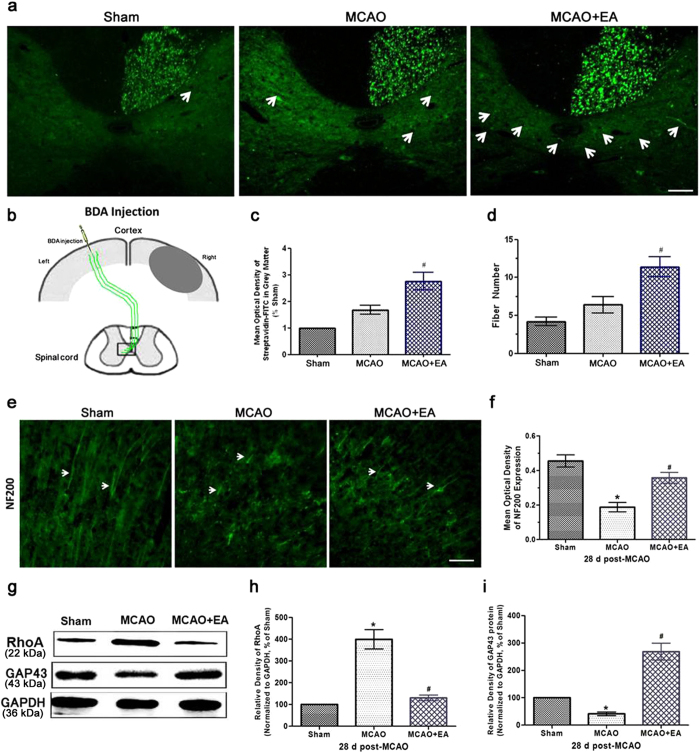
CST projection in the cervical cord and axon regeneration in the ischemic cortex at 28 d after stroke. (**a**) The normal rats exhibited unilateral CST innervation (Sham). Scarce BDA-labeled midline-crossing CST axons extended toward the ventral horn of the spinal gray matter on the denervated side of the cervical cord (C3-5) in the MCAO group. In contrast, significantly more BDA-positive CST axons were identified on the denervated side of the spinal cord in the MCAO + EA group. The BDA-labeled midline-crossing CST axons are green and are indicated by the arrows (scale bar = 50 μm). **(b)** A schematic diagram of the CST depicting the injection of BDA into the left (intact) cortex (scale bar = 100 μm). **(c)** Quantitative analysis of the mean optical density of the BDA-labeled midline-crossing CST axons, which extended toward the ventral horn of the spinal gray matter on the denervated side of the cervical cord (^#^*P* < 0.05 vs. MCAO group). **(d)** Statistical analysis of the number of fibers in the spinal gray matter on the denervated side of the cervical cord (^#^*P* < 0.05 vs. MCAO group). **(e)** Immunohistochemical staining for NF-200 was performed at 28 d post-MCAO in the ischemic penumbra (scale bar = 100 μm). **(f)** Bar graph indicating the relative optical intensity of NF-200 protein expression in the ischemic penumbra (*P < 0.05 vs. Sham group; ^#^*P* < 0.05 vs. MCAO group). **(g)** Western blot bands indicating RhoA and GAP43 expression in the ischemic penumbra at 28 d post-MCAO. The gels/blots were cropped for better showing figures. **(h)** Statistical analysis of the protein expression ratio of RhoA to GAPDH (*P < 0.05 vs. Sham group; ^#^*P* < 0.05 vs. MCAO group). **(i)** Statistical analysis of the protein expression ratio of GAP43 to GAPDH (*P < 0.05 vs. Sham group; ^#^*P* < 0.05 vs. MCAO group).

The axonal tracer biotionylated dextran amine (BDA) was injected into the intact (undamaged hemisphere) motor cortex at 14 d post-MCAO to label the descending axons in the intact hemisphere (Fig. 1b). As shown in Fig. 1a, the Sham group exhibited unilateral CST innervation at 28 d post-MCAO. In the MCAO group, scarce BDA-labeled midline-crossing CST axons extended toward the ventral horn of the spinal gray matter on the denervated side of the cervical cord, which served the affected limb. ANOVA showed a difference among the treatment groups [*F*(2,15) = 26.93, *P* < 0.001]. Bonferroni *post hoc* analyses showed that the amounts of BDA-positive CST axons on the middle and left sides of the spinal cord gray matter (C3–5) were significantly increased in the MCAO + EA group compared with the Sham and MCAO groups (*P* < 0.05, Fig. 1a and c). ANOVA showed a difference among the treatment groups [*F*(2,15) = 19.23, *P* < 0.001]. *Post hoc* analyses showed EA treatment significantly increased the number of these fibers detected ipsilateral to the injection site compared with that in the MCAO group (*P* < 0.05, Fig. 1a and d).

Axon damage and regrowth is typically assessed by immunostaining using an anti-NF-200 antibody. In the Sham group, long neuronal processes were detected that were immunoreactive for NF-200. The morphology of the NF-200-immunoreactive neuronal fibers was irregular and fragmented. ANOVA showed a difference among the treatment groups [*F*(2,15) = 45.38, *P* < 0.001]. *Post hoc* analyses showed NF-200 expression was less in the MCAO group compared with that in the Sham group at 28 d after reperfusion (*P* < 0.05). In addition, the NF-200 expression was significantly increased in the MCAO + EA group compared with the MCAO group at 28 d after reperfusion (*P* < 0.05, Fig. 1e and f).

The expression of axon growth-associated proteins was also evaluated. RhoA signaling plays an important role under pathological conditions, such as cerebral ischemia reperfusion injury and axon remodeling^30^. ANOVA showed a difference among the treatment groups [*F*(2,15) = 94.40, *P* < 0.001]. *Post hoc* analyses showed RhoA expression was increased in the MCAO group (*P*<0.05). However, its expression was inhibited after EA treatment at 28 d post-MCAO (*P* < 0.05, Fig. 1g and h). Growth-associated protein-43 (GAP43) is a pre-synaptic protein that regulates axon growth and guidance^31^. ANOVA showed a difference among the treatment groups [*F*(2,15) = 101.7, *P* < 0.001]. *Post hoc* analyses showed GAP43 protein expression was decreased in the MCAO group compared with the Sham group at 28 d post-MCAO (*P* < 0.05). EA treatment increased GAP43 protein expression compared with that in the MCAO group at 28 d post-MCAO (*P* < 0.05, Fig. 1g and i). These results demonstrated that EA treatment enhanced axon regeneration and the formation of new projections from the CST after stroke.

### EA inhibited PirB up-regulation in the ischemic penumbra after stroke

**Figure 2:**
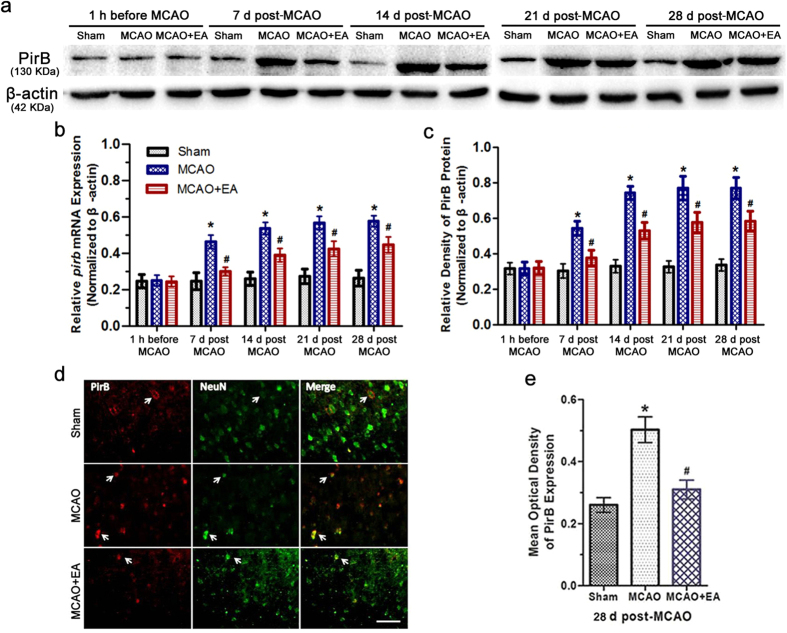
*pirb* mRNA and PirB protein expression in the ischemic penumbra after stroke. **(a)** Representative Western blot bands showing PirB expression in the ischemic penumbra. The gels/blots were cropped for better showing figures. **(b)** Bar graph showing the *pirb* mRNA levels calculated in the ischemic penumbra. The interaction is statistically significant. (*P < 0.05 vs. Sham group; ^#^*P* < 0.05 vs. MCAO group). **(c)** Bar graph showing the PirB protein levels calculated relative to β-actin. The interaction is statistically significant. (*P < 0.05 vs. Sham group; ^#^*P* < 0.05 vs. MCAO group). **(d)** Representative double-labeled (yellow) immunofluorescence staining of PirB (red)-positive and NeuN (green)-positive cells in brain sections at 28 d post-MCAO (scale bars = 50 μm). **(e)** Bar graph showed the expression of PirB in the ischemic penumbra at 28 d post-MCAO (*P < 0.05 vs. Sham group; ^#^*P* < 0.05 vs. MCAO group).

Expression of the *pirb* gene in the ischemic penumbra was examined via qRT-PCR, as Fig. 2b shown. There was a significant difference among the groups [*F*(8,75) = 11.72, *P* < 0.0001; *F*(2,75) = 161.26, *P* < 0.0001; *F*(4,75) = 40.12, *P* < 0.0001]. *Post hoc* analyses showed *pirb* mRNA level had no difference in every group at 1 h before MCAO surgery. The *pirb* mRNA level was higher in the MCAO groups than that in the Sham groups at 7, 14, 21 and 28 d post-MCAO (*P* < 0.05). In contrast, the *pirb* mRNA level was lower in the MCAO + EA groups than that in the MCAO groups at 7, 14, 21 and 28 d post-MCAO (*P* < 0.05).

Then, Western blot analysis revealed a low level of PirB protein expression in every group at 1 h before MCAO surgery. There was a significant difference among the groups [*F*(8,75) = 16.94, *P* < 0.0001; *F*(2,75) = 209.24, *P* < 0.0001; *F*(4,75) = 58.80, *P* < 0.0001]. *Post hoc* analyses showed PirB protein expression was higher in the MCAO groups than that in the Sham groups at 7, 14, 21 and 28 d after MCAO (*P* < 0.05). The result also showed the expression of PirB protein was lower in the MCAO + EA groups than that in the MCAO groups at 7, 14, 21 and 28 d after reperfusion (*P* < 0.05). (*P* < 0.05; Fig. 2a and c).

Next, we examined whether neurons in the ischemic penumbra region expressed PirB. We detected the presence of PirB/NeuN double-labeled neurons in this region at 28 d after reperfusion (Fig. 2d); ANOVA showed a difference among the treatment groups [*F*(2,15) = 32.57, *P* < 0.001]. *Post hoc* analyses showed the number of PirB-positive neurons in the MCAO group was greater than that in the Sham group at 28 d after reperfusion. In addition, the number of PirB-positive neurons in the MCAO + EA group was lower than that in the MCAO group at 28 d after reperfusion (Fig. 2e and f). These results suggested that EA treatment inhibited PirB expression in the ischemic penumbra after reperfusion.

### *pirb* mRNA down-regulation facilitated neurite growth in cortical neurons exposed to oxygen-glucose deprivation (OGD) *in vitro*

**Figure 3:**
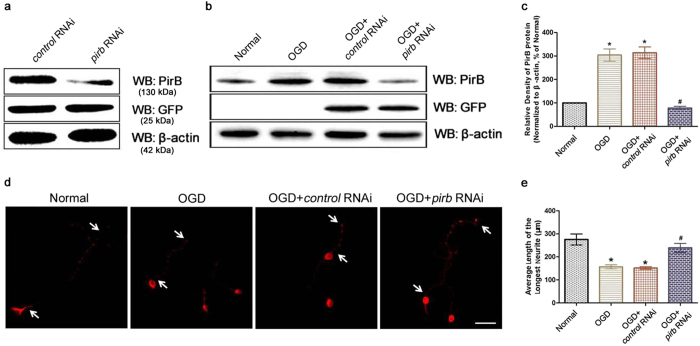
Effects of PirB knockdown on neurite regrowth in cortical neurons exposed to OGD. (**a**) RNAi-mediated silencing of PirB in HEK293 cells. PirB (upper panel), GFP (middle panel) and β-actin (lower panel) expression was detected in extracts by Western blot analysis at 24 h after transfection. The gels/blots were cropped for better showing figures. **(b)** RNAi-mediated knockdown of PirB in primary cortical neurons after OGD injury. PirB (upper panel), GFP (middle panel) and β-actin (lower panel) expression was detected in cell extracts via Western blot analysis at 24 h after transfection in each group. The gels/blots were cropped for better showing figures. **(c)** Bar graph showing a comparison of the protein levels of PirB and β-actin, as determined by Western blot analysis (*P < 0.05 vs. Normal group; ^#^*P* < 0.05 vs. OGD group). **(d)** Immunofluorescence staining was performed to measure neurite length at 72 h after transfection. The arrowheads indicate the longest neurites (scale bars = 50 μm). **(e)** Statistical analysis of the average length of the longest neurite in each group based on three independent experiments (*P < 0.05 vs. Normal group; ^#^*P* < 0.05 vs. OGD group).

We used HEK293 cells to determine the efficacy of the RNA interference (RNAi)-mediated reduction in PirB expression. At 24 h after transient transfection, Western blot analysis indicated that the GFP expression levels were similar between the *control* RNAi and *pirb* RNAi groups. However, the PirB expression level in the *pirb* RNAi group was significantly lower than that in the *control* RNAi group (Fig. 3a). An *in vitro* OGD model was used to evaluate the efficacy of *pirb* RNAi following ischemic injury. At 24 h post-OGD, the Western blot analysis revealed that PirB expression in the OGD and control RNAi groups was increased compared with that in the uninjured group (*P* < 0.05). However, its expression was significantly decreased in the *pirb* RNAi group compared with the OGD and *control* RNAi groups (*P* < 0.05, Fig. 3b,c).

The neurites of cortical neurons were then examined to assess the effects of *pirb* RNAi on neurite growth. Under normal culture conditions, cortical neurons contained a single long axon *in vitro*. ANOVA showed a difference among the treatment groups [*F*(3,20) = 21.69, *P* < 0.001]. *Post hoc* analyses showed the extension of cultured cortical neuronal axons was significantly reduced at 72 h after exposure to OGD (*P* < 0.05). However, the mean axon length in the *pirb* RNAi group was longer than those in the OGD and *control* RNAi groups (*P* < 0.05, Fig. 3d and e). These results indicated that the reduction in PirB expression enhanced neurite growth after OGD injury *in vitro*.

### EA increased the miR-181b level in the rat ischemic penumbra after stroke

**Figure 4:**
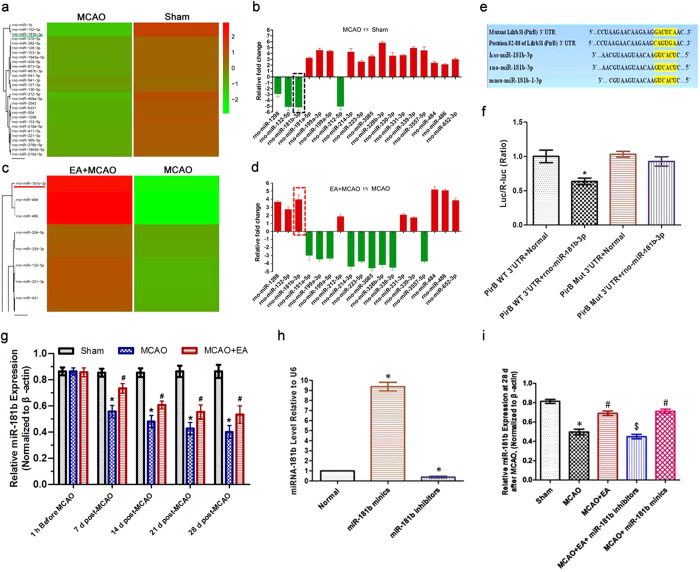
EA increased the miR-181b level in ischemic penumbra after stroke. **(a,c)** Hierarchical cluster microarray analysis of the miRNAs with altered levels in the penumbra region in each group at 28 d post-MCAO. The color code in each heat map is linear, with green indicating the lowest level and red representing the highest level. **(b,d)** Among the remarkably changed 20 miRNAs, the expression of miR-181b was down-regulated in the MCAO group compared with the Sham group. In contrast, its expression was dramatically increased in the MCAO + EA group compared with the MCAO group. **(e)** The sequence of rat miR-181b and the predicted binding sites within the PirB 3′-UTR in different species are shown. The sequence of the PirB 3′-UTR mutant used for reporter assay is also shown. **(f)** Luciferase reporter constructs containing the PirB wild-type (WT) 3′-UTR or a PirB mutant (Mut) 3′-UTR of the *pirb* gene were co-transfected with miR-1b1b or an empty vector into HEK293T cells, and luciferase activity was assayed (*P < 0.05 vs. PirB WT 3′-UTR + Normal group). **(g)** Bar graph showing the miR-181b levels calculated in the rat ischemic penumbra. (*P < 0.05 vs. Sham group; ^#^*P* < 0.05 vs. MCAO group) **(h)** Evaluation the effects of miR-181b mimics and inhibitors on the miR-181b level in primary neurons *in vitro*. Bar graph showing the relative expression of miR-181b normalized to the expression of the small RNA U6B (*P < 0.05 vs. Normal group). **(i)** Bar graph showing the relative expression of miR-181b in the ischemic penumbra in each group (*P < 0.05 vs. Sham group; ^#^*P* < 0.05 vs. MCAO group; ^$^*P* < 0.05 vs. MCAO + EA group).

Differential expression of miRNAs was evaluated in the rat ischemic penumbras in the Sham, MCAO and MCAO + EA groups at 28 d post-MCAO. The miRNA microarray analysis resulted in the significant change of 20 differential miRNAs in the ischemic penumbra of the rats in each group. Among these 20 miRNAs, miR-181b expression was markedly down-regulated in the MCAO group compared with the Sham group (Fig. 4a and b). In contrast, its expression was increased dramatically in the MCAO + EA group compared with the MCAO group (Fig. 4c and d). Intriguingly, using computational miRNA TargetScan analysis (http://www.targetscan.org) to search for targets of miR-181b, we found that the 3′-UTR of *pirb* mRNA contains two putative miR-181b target sites (Fig. 4e). To determine whether the *pirb* mRNA is potentially regulated by miR-181b, we constructed a reporter plasmid by inserting the cDNA corresponding to the 3′-UTR of *pirb* mRNA into the 3′region of the firefly luciferase gene. Cells were co-transfected with the firefly luciferase target reporter plasmid containing the *pirb* 3′-UTR (with either wild-type or mutated miR-181b binding sites), a Renilla luciferase control reporter, and miR-181b mimics or a negative control. ANOVA showed a difference among the treatment groups [*F*(3,20) = 7.614, *P* < 0.001]. *Post hoc* analyses showed miR-181b caused a significant decrease in luciferase activity (*P* < 0.05; Fig. 4f), whereas they did not alter the luciferase activity in cells transfected with reporter constructs containing a mutated miR-181b 3′-UTR. These results suggest that miR-181b may directly regulate *pirb* mRNA expression and have a very important function in the treatment of cerebral ischemic-reperfusion injury using EA.

Next, miR-181b expression in the ischemic penumbra was examined. There was a significant difference among the groups [*F*(8,75) = 13.85, *P* < 0.0001; *F*(2,75) = 190.1, *P* < 0.0001; *F*(4,75) = 50.20, *P* < 0.0001]. *Post hoc* analyses showed miR-181b level had no remarkable difference in every group at 1 h before MCAO surgery (*P* > 0.05). Its level was lower in the MCAO group than that in the Sham group at 7, 14, 21 and 28 d after reperfusion (*P* < 0.05). In contrast, the miR-181b level was increased in the MCAO + EA group compared with that in the MCAO group at 7, 14, 21 and 28 d after reperfusion (*P* < 0.05; Fig. 4g).

**Figure 5:**
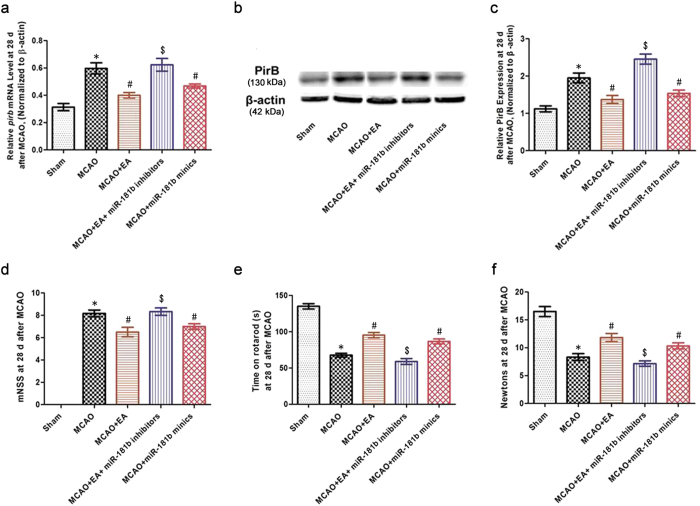
EA enhanced rehabilitation through miR-181bdirect targeting PirB after ischemic stroke. **(a)** Bar graph showing the *pirb* mRNA levels in the rat ischemic penumbra at 28 d post-MCAO. **(b)** Representative Western blot bands showed PirB expression in the ischemic penumbras at 28 d post-MCAO. The gels/blots were cropped for better showing figures. **(c)** Bar graph showing the PirB protein levels relative to β-actin. **(d–f)** mNSS calculations, the rota-rod and grip strength tests were performed at 28 d post-MCAO. (*P < 0.05 vs. Sham group; ^#^*P* < 0.05 vs. MCAO group; ^$^*P* < 0.05 vs. MCAO + EA group).

Furthermore, mimics or inhibitors of miR-181b were transfected into the primary neuron cultures. ANOVA showed a difference among the treatment groups [*F*(2,15) = 415.7, *P* < 0.001]. *Post hoc* analyses showed miR-181b level in the miR-181b mimics group was higher than that in the Normal group (*P* < 0.05; Fig. 5h). In contrast, its level was lower in the miR-181b inhibitors group compared with that in the Normal group (*P* < 0.05; Fig. 5h). Next, miR-181b mimics or miR-181b inhibitors were transfected into cerebral cortex neurons in the ischemic penumbra. ANOVA showed a difference among the treatment groups [*F*(4,25) = 42.31, *P* < 0.001]. *Post hoc* analyses showed that EA treatment or miR-181b mimic transfection increased the miR-181b level at 28 d post-MCAO (*P*<0.05; Fig. 5i); however, this effect was reversed by miR-181b inhibitor transfection (*P* < 0.05; Fig. 5i). These results demonstrate that EA can regulate the miR-181b level in the ischemic penumbra after stroke.

### EA enhanced neurobehavioral functional recovery through miR-181b-mediated *pirb* gene silencing after ischemic stroke

We examined the effects of miR-181b on *pirb* mRNA and PirB protein expression in the ischemic penumbra after stroke. ANOVA showed a difference among the treatment groups for *pirb* mRNA test [*F*(4,25) = 16.60, *P* < 0.001] and for PirB protein test [*F*(4,25) = 22.52, *P* < 0.001]. *Post hoc* analyses showed that EA treatment or miR-181b mimic transfection decreased *pirb* mRNA and PirB protein expression at 28 d post-MCAO (*P* < 0.05; Fig. 5a–c); however, these effects were reversed by miR-181b inhibitor transfection (*P* < 0.05; Fig. 5a–c). These results demonstrated that miR-181b targeted *pirb* mRNA to regulate PirB expression.

Moreover, neurobehavioral function was evaluated. ANOVA showed a difference among the treatment groups for mNSS test [*F*(4,25) = 130.0, *P* < 0.001], for rota-rod test [*F*(4,25) = 69.40, *P* < 0.001] and for grip strengths test [*F*(4,25) = 29.33, *P* < 0.001]. The mNSS of the MCAO + EA group and MCAO+miR-181b mimics group were significantly lower than that of the MCAO group at 28 d after reperfusion (*P* < 0.05); this effect was reversed by miR-181b inhibitor transfection (*P* < 0.05; Fig. 5d). In addition, the times on the rota-rod and grip strengths were increased in the MCAO + EA group and MCAO+miR-181b mimics group compared with MCAO group at 28 d after reperfusion (*P* < 0.05). However, miR-181b inhibitor transfection altered the effects of EA or the miR-181b mimics on the rota-rod and grip strength performances (*P* < 0.05; Fig. 5e and f). These results suggested that EA enhanced neurobehavioral functional recovery by promoting the targeting of *pirb* mRNA by miR-181b after ischemic stroke.

### MiR-181b targeted *pirb* mRNA to regulate PirB and its downstream signaling molecules after ischemic-reperfusion injury

**Figure 6:**
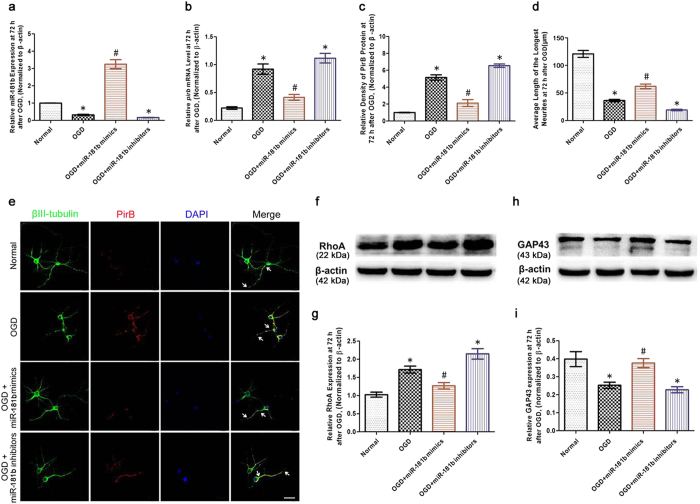
The effects of miR-181b on PirB, RhoA and GAP43 expression after OGD injury. (**a)** Bar graph showing the miR-181b levels in neurons at 72 h after OGD injury. **(b)** Bar graph showing the *pirb* mRNA levels in neurons at 72 h after OGD injury. **(c)** Statistical analysis of PirB expression in neurons at 72 h after OGD injury. **(d)** Statistical analysis of the average length of the longest neurite at 72 h after OGD injury. **(e)** Immunocytochemistry staining demonstrating PirB expression and neurite growth in neurons at 72 h after OGD injury. Representative triple-label immunofluorescence staining with βIII-tubulin (green), PirB (red) and DAPI (blue). The neuritis were indicated by the arrows (scale bars = 50 μm). **(f,h**) Representative Western blot bands showing RhoA and GAP43 expression in neurons at 72 h after OGD injury. The gels/blots were cropped for better showing figures. **(g,i)** Bar graph showing the RhoA and GAP43 expression levels relative to β-actin. (*P < 0.05 vs. Normal group; ^#^*P* < 0.05 vs. OGD group).

To determine whether miR-181b is associated with the expression of PirB and its downstream signaling molecules after ischemic-reperfusion injury, the effects of miR-181b mimics and miR-181b inhibitors on miR-181b expression in a model of OGD were detected *in vitro*. ANOVA showed a difference among the treatment groups [*F*(3,20) = 118.3, *P* < 0.001]. *Post hoc* analyses showed the miR-181b level was lower in the OGD group compared with the Normal group at 72 h after OGD injury (*P* < 0.05; Fig. 6a). In addition, significantly increased miR-181b expression was observed in the OGD+miR-181b mimics group compared with the OGD group (*P* < 0.05; Fig. 6a). In contrast, decreased miR-181b expression was detected in the OGD+miR-181b inhibitors group compared with the Normal group (*P* < 0.05; Fig. 6a).

Next, we assessed the effects of miR-181b mimics and miR-181b inhibitors on *pirb* mRNA in the model of OGD. ANOVA showed a difference among the treatment groups for *pirb* mRNA test [*F*(3,20) = 37.07, *P* < 0.001]. Decreased *pirb* mRNA was observed in the OGD+miR-181b mimics group compared with the OGD group at 72 h after OGD injury (*P* < 0.05; Fig. 6b and c). In contrast, the expression was higher in the OGD group and OGD+miR-181b inhibitors group compared with the Normal group at 72 h after OGD injury (*P* < 0.05; Fig. 6b and c). Moreover, the PirB level and neurite outgrowth were evaluated at 72 h after OGD injury. As shown in the Fig. 6d and e, ANOVA showed a difference among the treatment groups for PirB protein test [*F*(3,20) = 86.14, *P* < 0.001]. The analyses showed that transfection of miR-181b mimics into neurons led to a decrease in PirB expression (*P* < 0.05); in contrast, transfection of miR-181b inhibitors significantly increased its expression (*P* < 0.05). In addition, ANOVA showed a difference among the treatment groups [*F*(3,20) = 126.2, *P* < 0.001]. *Post hoc* analyses showed the longest neurite length was significantly inhibited in the OGD group compared with the Normal group *(P* < 0.05). However, the longest neurite length was almost completely restored in the OGD+miR-181b mimics group compared with the OGD group (*P* < 0.05). In contrast, the neurite length was significantly reduced in the OGD+miR-181b inhibitors group compared with the OGD+miR-181b mimics group (*P* < 0.05). These findings suggested that miR-181b up-regulation enhanced neurite growth by inhibiting PirB expression after OGD injury.

**Figure 7:**
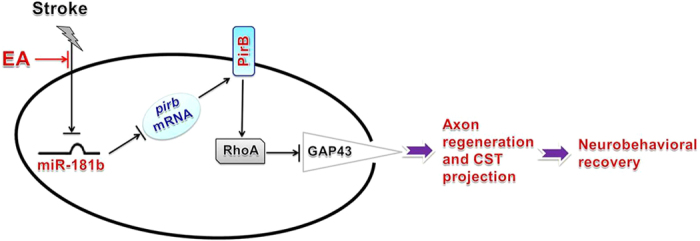
Hypothetical model depicting epigenetic mechanisms underlying the therapeutic effects of EA against ischemic stroke. EA enhances neurobehavioral functional recovery from ischemic stroke through a series of complex processes, which may include increasing the miR-181b level, decreasing PirB expression, and regulating the levels of PirB downstream signaling molecules (such as RhoA and GAP43). The findings of this study indicate that EA exerts its therapeutic effects by inducing epigenetic changes to directly regulate its targets, such as the miR-181b/PirB/RhoA/GAP43 axis.

Furthermore, Western blot analysis tested RhoA expression. ANOVA showed a difference among the treatment groups [*F*(3,20) = 23.49, *P* < 0.001]. *Post hoc* analyses revealed that RhoA expression increased in the OGD group compared with the Normal group (*P* < 0.05; Fig. 6f and g). However, its expression decreased in the OGD+miR-181b mimics group compared with the OGD group (*P* < 0.05; Fig. 6f and g). In contrast, RhoA expression increased in the OGD+miR-181b inhibitors group compared with the OGD+miR-181b mimics group (*P* < 0.05; Fig. 6f and g). Furthermore, Western blot analysis tested GAP43 expression. ANOVA showed a difference among the treatment groups [*F*(3,20) = 10.08, *P* < 0.001]. *Post hoc* analyses revealed GAP43 expression was reduced in the OGD group compared with the Normal group (*P* < 0.05; Fig. 7h and i). In contrast, GAP43 expression increased in the OGD+miR-181b mimics group compared with the OGD group (*P* < 0.05; Fig. 6h and i), and decreased in the OGD+miR-181b inhibitors group compared with the OGD+miR-181b mimics group (*P* < 0.05; Fig. 6h and i). These results suggested that miR-181b regulated the expression of PirB and its downstream signaling molecules, thereby restricting neurite growth after ischemic injury. This may be the mechanism underlying the EA-produced rehabilitation of axon regeneration, CST projection and neurobehavioral functional recovery, as shown in Fig. 7.

## Discussion

Acupuncture has been used for stroke recovery in East Asia for centuries. The positive effects of acupuncture have been reported in studies of its application to treat neurological disorders^7^,^32^. Specifically, the use of acupuncture or EA for the treatment of stroke has been shown to be effective for improving brain damage and motor function^8^,^33^. However, its effect and mechanism on motor rehabilitation remain to be clearly elucidated in animal studies^34^,^35^. After transient focal cerebral ischemia injury, the extents of brain and behavioral recovery are influenced by neural circuit plasticity, which includes axon regeneration and remodeling, particularly rewiring and the formation of new projections from the CST after stroke^12^,^13^. In the present study, we demonstrated that EA treatment increased the number of BDA-positive CST axons projecting from the undamaged CST to the middle or left side of the spinal cord gray matter (C3–5) at 28 d post-MCO. We also found that EA treatment enhanced NF-200 and GAP43 expression, whereas it inhibited RhoA expression, at 28 d post-MCAO. Previous studies have demonstrated that neurons in the intact cortex can extend their axons into damaged regions, which are amenable to motor functional recovery^13^. Furthermore, the sprouting and growth of intracortical axons plays important roles after cerebral ischemia-reperfusion injury^15^,^16^. Other researchers have suggested that neurofilaments expressed in axons are among the most important structural substrates of the axonal cytoskeleton, which is necessary for axonal maintenance and regeneration^36^,^37^. These results support the view that EA treatment exerts rehabilitation effect by enhancing axon regeneration and CST projection after stroke.

PirB is a type I transmembrane glycoprotein consisting of an extracellular portion that contains six Ig-like domains, a hydrophobic transmembrane segment and a cytoplasmic portion that contains three immunoreceptor tyrosine-based inhibitory motifs (ITIMs) and one ITIM-like sequence^17^. Atwal *et al*. first found that the inhibition of PirB activity using a neutralizing antibody alleviated the suppression of axon growth and growth cone collapse and demonstrated that PirB is a novel functional receptor for Nogo-66, MAG, and Omgp for inhibiting axon growth and regeneration^17^. Fujita *et al*. further found that the binding of PirB to Nogo, OMgp or MAG induced downstream signals, for example, activation and delivery of the inhibitory signal for neurite outgrowth^18^. Our previous study has revealed that neuronal PirB expression is up-regulated in the ischemic penumbras of mice from 24 h to 7 d after reperfusion^19^. In this study, we found that the *pirb* mRNA and PirB protein expression were decreased following EA treatment in the ischemic penumbra from 7 to 28 d after stroke. The results provided the evidence that EA administration can effectively inhibit PirB up-regulation in the ischemic penumbra after stroke. Recent studies have demonstrated that PirB contributes to stabilization of the adult neuronal circuitry and motor function by inhibiting axon regeneration and synaptic plasticity^38^,^39^. Moreover, *pirb* knockout mice exhibit increased crossed projections of CST axons and improved motor performance^15^. The down-regulation of PirB expression in cortical neurons has been shown to result in increased neurite outgrowth^40^. These previous findings are consistent with our current study. Thus, it is plausible to deduce that the significant up-regulation of PirB expression in the ischemic penumbra may be one of crucial “troublemaker” that exacerbates brain damage and restricts motor recovery following cerebral ischemia-reperfusion injury. Moreover, EA enhanced axon regeneration via the down-regulation of PirB expression, indicating that PirB is a novel therapeutic target of EA for promoting neurobehavioral functional recovery following ischemic stroke.

Recently, a large number of reports have been published on the involvement of miRNAs in the response to EA therapy against cerebral ischemia reperfusion injury^40^. In particular, the relevance of miR-181b function to the organization of neural connections has been reported and identified. MiR-181b, a member of the miR-181 family, is expressed at intriguingly high levels in the retina and brain areas associated with motor function^29^. Furthermore, increasing evidence shows that miR-181b governs axon specification and growth by regulating its target signaling molecules and plays an important role in the organization of neural connections^29^,^41^. However, the role of miR-181b in the ischemic penumbra in association with EA treatment for stroke remains to be elucidated. Interestingly, miRNA microarray analysis revealed that miR-181b expression was significantly decreased in the ischemic penumbra at 28 d post-MCAO, whereas EA treatment enhanced its expression. A similar changing trend was identified by qRT-PCR after reperfusion. Furthermore, these results provide the first evidence that EA directly regulates miR-181b expression following administration of miR-181b mimics or miR-181b inhibitors. These results suggest that an increase in the brain-specific miR-181b level in the ischemic penumbra may be directly responsible for the mechanical repair of brain tissue, and they also imply that the rehabilitative effects of EA administration may include promoting miR-181b expression after cerebral ischemic injury.

To further elucidate the underlying molecular mechanisms of EA-produced neurobehavioral functional recovery, we searched for a possible relationship between miR-181b and *pirb* mRNA, which may be the miR-181b target that participates in this process. Computational miRNA target analysis revealed that the *pirb* 3′-UTR was a predicted target of miR-181b. Luciferase assay further demonstrated that PirB was likely a direct target of miR-181b. The results of these investigations increase the understanding of the mechanisms underlying the up-regulation of miR-181b expression and down-regulation of PirB expression by EA in the ischemic penumbra. Our results also showed that EA treatment and miR-181b mimic administration had similar effects on *pirb* mRNA and PirB protein expression. In contrast, the miR-181b inhibitors increased the *pirb* mRNA and PirB protein levels. These findings verify that PirB is a direct target of miR-181b and demonstrate that EA enhances neurobehavioral performance recovery through miR-181b-mediated*pirb* gene silencing after ischemic stroke.

To explore the mechanisms of the miR-181b-mediated regulation of PirB expression and neurite growth, an OGD model was used. To the best of our knowledge, our study is the first to demonstrate a decrease in the miR-181b level at 72 h after OGD injury. Moreover, the decreased miR-181b expression inhibited neurite growth after OGD injury, which could be rescued by miR-181b mimics. One possible explanation for these findings is that miR-181b increased *pirb* mRNA and PirB protein expression to regulate the expression of downstream signaling molecules, such as RhoA and GAP43. These results are consistent with those of the above *in vivo* study, in addition to a previous report demonstrating that PirB inhibition promotes the regeneration of hypoxic-ischemic-damaged axons and that this inhibitory signal may be transduced via the Rho-ROCK signaling pathway^29^,^41^. Moreover, GAP43 is another downstream molecule of PirB that facilitates axonal sprouting and outgrowth^16^. These results indicate that miR-181b targets *pirb* mRNA to regulate the expression of PirB and its downstream signaling molecules after ischemic-reperfusion injury.

In conclusion, as shown in Fig. 7, this study first provides and verifies the solid evidences that EA treatment enhances neurobehavioral functional recovery through miR-181b targeting PirB after ischemic stroke, suggesting that miR-181b and PirB act as key regulators of axon regeneration and CST projection after cerebral ischemic-reperfusion injury. These findings indicate that EA exerts its therapeutic effects on stroke by regulating epigenetic changes to directly act on its targets, such as the miR-181b/PirB/RhoA/GAP43 axis, which may be a novel mechanism underlying EA treatment against ischemic stroke.

## Materials and Methods

### Animals

Male Sprague-Dawley rats (280–300 g) were provided by the Experimental Animal Center of the Fourth Military Medical University and were housed under controlled conditions, including a 12-h light/dark cycle, a temperature of 21 ± 2 °C and humidity of 60–70%, for at least one week prior to receiving drug treatment or surgery. The animals were allowed free access to standard rodent food and tap water. The experimental protocol used in this study was conducted according to the Guidelines for Animal Experimentation of the Fourth Military Medical University and was approved by the Ethics Committee for Animal Experimentation of the Fourth Military Medical University (Xi’an, Shaanxi province, China).

### Middle cerebral artery occlusion (MCAO) model

The focal cerebral ischemic model of rat was induced by MCAO as described previously^42^. Briefly, the rats were anesthetized with 2% chloral hydrate. The right middle cerebral artery of the rats was occluded using a 3-0 nylon monofilament suture (Ethicon Nylon Suture, Ethicon Inc., Sukagawa, Japan). Regional cerebral blood flow (rCBF) was monitored using a disposable microtip fiberoptic probe (diameter, 0.5 mm) connected through a master probe to a computerized laser Doppler main unit (PeriFlux 5000, Perimed AB, Sweden). Rats retaining >20% of baseline perfusion during ischemia were excluded. The temporalis muscle temperature was monitored and was maintained at 37.0 °C–37.5 °C by surface heating or cooling during surgery. After 120 min of ischemia, reperfusion was done. At 120 min after reperfusion, the rats received EA treatment. After that, rats were then allowed to recover before being returned to their home cages.

### EA treatment

The animals were anesthetized with 2% chloral hydrate, and then the “Baihui (GV 20)” acupoint^42^, which is located at the intersection of the sagittal midline and the line linking the two rat ears, was stimulated electrically at an intensity of 1-2 mA and dense-disperse frequency of 2/10 Hz. A fine needle (0.5 mm in diameter) placed at GV 20 was connected to one electrode of a HWATO electronic acupuncture treatment instrument (model No. SDZ-V, Suzhou Medical Appliances Co., Ltd., Suzhou, China). Individual EA sessions were administered for 30 min per day for 5 successive days, followed by 2 days of rest, for a period of 4 weeks. The core temperatures of the rats receiving the anesthesia and EA treatment were maintained at 37.0 ± 0.5 °C by surface heating or cooling (Spacelabs Medical Inc.).

### Retrograde tracing of the CST

Rats which had been operated successfully were randomly divided into the following groups: Sham, MCAO and MCAO + EA group (n = 6 for each group). After reperfusion, the rats received EA treatment. At 14 d after reperfusion, the rats were anesthetized using 2% chloral hydrate, and a large craniotomy was performed to expose the left hemisphere of the brain. Surgeries were performed using a Kopf stereotaxic setup and procedures adapted from previous study^13^. The cortex overlying the injection area was exposed by removing the skull using a dental drill. Then, BDA (NeuroTrace Neuronal Tracer Kit, Molecular Probes, Eugene, OR) was injected using a Nanoject injector (Drummond Scientific, Broomall, PA) at different sites, with the delivery of 100 nl BDA at three depths per site (0.5, 1.0, and 1.5 mm deep from the cortical surface). The injection site coordinates were as follows (in mm): 3.8 L/1.2A, 3.4 L/0.7A, 3.4 L/0.2A, 3.6 L/0.3P, 3.4 L/1.3P, 2.2 L/1/3P, 2.2 L/2.3P, 2.2 L/2.8P, and 3.0 L/3.3P. These coordinates are described with respect to bregma. Each 100-nl injection was performed over a 1-min period, such that the time required to inject each site was approximately 4–5 min. After completion of the injections, the scalp was sutured. The body temperature was maintained at 37 °C using a warming blanket.

After 28 d after reperfusion, the rats were deeply anesthetized and perfused, and coronal brain sections were obtained. Selected sections through the cervical region of the spinal cord were prepared as described by Oswald Steward. BDA was stained using Streptavidin-FITC (1:100, eBioscience). BDA-labeled axons in the dorsal CST ipsilateral to the cortex of origin (left hemisphere) were counted in four sections in the cervical enlargement. BDA-positive fibers were examined in the cervical enlargement of the spinal cord. Images were captured using an Olympus BX-60 fluorescence microscope (Olympus Corporation, Shinjuku, Tokyo, Japan).

### Quantitative real-time-polymerase chain reaction (qRT-PCR)

Rats which had been operated successfully were randomly divided into the following groups: Sham, MCAO, MCAO + EA group (n = 6 for each group). After reperfusion, the rats received EA treatment. At 1 h before MCAO, 7 d post-MCAO, 14 d post-MCAO, 21 d post-MCAO, 28 d post-MCAO, the rats were deeply anesthetized and obtained brain tissue. The *pirb* mRNA and miR-181b expression were tested by qRT-PCR analysis. The ischemic penumbra of the cerebral cortex at the Bregma level +2.0 to −5.0 mm was harvested as previously described^42^. The total RNA was isolated from each sample using RNAiso Plus (TaKaRa) according to a standard protocol and subsequently quantified. cDNA was synthesized using the Superscript First-Strand Synthesis Kit (Promega, Madison, WI) according to the manufacturer’s instructions. PCR was performed under the following thermal cycling conditions: one cycle at 94 °C for 5 min; 25 cycles at 94 °C for 30 s, 55 °C for 60 s, 72 °C for 30 s; and one cycle at 72 °C for 10 min. The following primers were designed by TaKaRa Corp.: PirB (Sense 5′-CATGTCATGGGGACTGCCCT-3′, Antisense 5′-CCAGAGTCATTCCAAGACAGAGC-3′); β-actin (Sense 5′-TCAGGTCATCACTATCGGCAAT3-′, Antisense 5′-AAAGAAAGGGTGTAAAACGCA-3′). To perform qRT-PCR of miR-181b, a small RNA fraction was isolated with a mirVana miRNA Isolation Kit (Genetimes Technology, Inc.), and 200 pg miRNA were used for each reaction. qRT-PCR analysis was performed using an Agilent Technologies Stratagene Mx3000P Real-Time PCR system (Genetimes Technology, Inc.) with real-time SYBR Green PCR technology. The threshold cycle (Ct) value was determined using the automatic baseline determination feature of Stratagene MX3000. Each sample was tested in triplicate. The samples were obtained from three independent experiments and were analyzed for relative gene expression using the 2-ΔΔCT method. The efficiency of each primer was determined and used to quantify the expression level as follows:


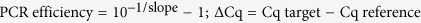



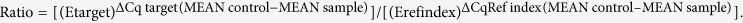


### Western blotting analysis

To determine the expression of PirB, RhoA, GAP43, GAPDH and β-actin in the ischemic penumbra of rats *in vivo* or in the neurons *in vitro* studies, Western blotting analyses were performed at different time-points after ischemia/reperfusion. For PirB protein long term expression assessments: The rats which had been operated successfully were randomly divided into 15 groups (n = 6 per group): Sham group (5 groups), MCAO group (5 groups) and MCAO + EA group (5 groups). The animals in every group were decapitated at different time points after reperfusion (at 1 h before MCAO, 7 d post-MCAO, 14 d post-MCAO, 21 d post-MCAO and 28 d post-MCAO). For RhoA and GAP43 protein expression assessments: The rats from the above group were used at 28 d post-MCAO. The total protein from each ischemic penumbra was acquired using an extraction kit (KeyGEN, Nanjing, China) on ice. The following primary antibodies and the secondary antibodies were used. The signals were detected using an ECL kit (Pierce). The relative changes in protein expression were expressed as the ratio of the integrated optical density of the target protein band to that of β-actin or GAPDH. The experiments were performed independently in triplicate. The full-length gels and blots are included in the supplementary information.

### Immunofluorescence staining

First, PirB/NeuN double immunofluorescence analyses in the ischemic penumbra of rats were performed on the Sham, MCAO and MCA + EA groups at 28 d after ischemia/reperfusion. Rabbit monoclonal anti-PirB antibody (1:100, Abcam, Cambridge, England) and mouse monoclonal anti-NeuN antibody (1:500, Millipore, Temecula, CA, USA) were used. The secondary antibodies were FITC-labeled goat anti-mouse IgG (1:2000, Molecular Probes, USA) and Alexa Fluor 594-conjugated anti-rabbit IgG (1:1000, Molecular Probes). Alternatively, immunofluorescence staining for NF200 in the ischemic penumbra of rats was performed on the Sham, MCAO and MCAO + EA groups at 28 d after ischemia/reperfusion. Staining was performed using mouse monoclonal anti-NF200 antibody (1:500, Abcam) FITC-labeled goat anti-mouse IgG secondary antibody (1:2000, Molecular Probes). Finally, the sections were examined and images were captured using an Olympus BX-60 fluorescence microscope (Olympus Corp., Shinjuku, Tokyo, Japan).

### Expression constructs and RNAi

The short interfering RNA (siRNA) expression vector pLenR-GIP-GFP was used, and the PirB (Lilrb3, NM_031713.1) siRNA sequence was 5′GCTGGAAAGTTATGTGAATGC3′. Western blot analysis confirmed the knockdown of endogenous PirB in the transfected HEK293 cells and primary cortical neurons. Briefly, HEK293 cells were transiently transfected with a *control* RNAi construct or PirB expression construct, which also expressed GFP. The expression of PirB, GFP and β-actin in cell was determined by Western blot analysis using the corresponding specific antibodies (anti-PirB rabbit monoclonal antibody, 1:1000, Abcam; anti-GFP rabbit monoclonal antibody, 1:1000, Abcam; and anti-β-actin rabbit monoclonal antibody, 1:2000, Abcam, respectively) at 24 h after transfection. The secondary antibodies were used as previously described.

### Primary cortical neurons culture and oxygen glucose deprivation (OGD) model

Primary cortical neurons were cultured as previously described^43^. In brief, neurons were isolated from SD rat embryos (E18.5 d), washed with D-Hank’s solution three times under sterile conditions and seeded at a density of 1 × 10^5^ cells/cm^2^ on plates coated with poly-L-lysine (50 mg/mL) (Sigma, USA). The cells were cultured in Neurobasal medium (Gibco, Invitrogen Corp., USA), which was supplemented with 2% B27, 1% glutamine, and 1% penicillin/streptomycin (Sigma, USA), at 37 °C in a humidified incubator in air that contained 5% CO_2_. The purity of the neurons was determined via immunocytochemistry for β-III-tubulin (1:250; Millipore) at 7 d after seeding, which indicated that 95% of the culture cells were positive for β-III-tubulin (data not shown).

The OGD model was used as previously described^44^. In brief, cortical neurons were cultured in Neurobasal medium that lacked glucose (Invitrogen, Carlsbad, CA, USA) in a humidified incubator which contained an anoxic gas mixture (5% CO_2_ and 95% N_2_) at 37 °C for 1 h; the neurons were subsequently cultured in normal medium. The cells were transiently transfected with the *control* RNAi construct or the *pirb* RNAi construct. The cultures were divided into groups as follows: Normal, OGD, OGD+*control* RNAi and OGD+*pirb* RNAi group.

To evaluate the effect of *pirb* RNAi on PirB expression and neurite growth after OGD injury, the expression of PirB, GFP and β-actin in the extracts was determined via Western blot analysis at 24 h after transfection. Then, cells were subjected to immunocytochemistry staining at 72 h after transfection. The cells were washed after fixation in 4% PFA and subsequently stained with a rabbit monoclonal anti-Tau antibody (1:1000, Abcam). The measurement of neurite length was performed as follows. Five randomly selected fields of view on the slides were photographed using a phase-contrast Olympus IMT2 microscope and an F-View camera. The average length of the longest neurites was measured as the staining for Tau (1:500; Millipore), which labels axons. The average lengths of the longest neurites from three independent experiments were analyzed using Student’s t-test, as previously described^44^.

### MicroRNA microarray analysis

Rats which had been operated successfully were randomly divided into the following groups: Sham, MCAO, MCAO + EA group (n = 6 for each group). After reperfusion, the rats received EA treatment. At 28 d post-MCAO, RNA was isolated from each cerebral ischemic penumbra using the mirVana miRNA Isolation Kit (Ambion, USA). The miRNA microarray was implemented according to MicroRNA Expression Profiling Assay Guide (Illumina Inc., USA). After adding a stretch of poly A tail to the 3′-end of each sequence in the 1000 ng intact total RNA sample. Then the biotinylated cDNAs were linked with miRNA specific oligos in an assay specific extension plate. The oligos were hybridized to the cDNA, mis-hybridized and excess oligos were washed away. In this process, the BeadChips were hybridized using the hybridization chamber. The BeadChips were hybridized overnight in the Illumina hybridization oven, with a temperature ramp from 60 °C to 45 °C. The Illumina BeadArray Reader (Illumina Inc.) used a laser to excite the fluor of the single-base extension product on the beads of the BeadChip sections.

### Dual-luciferase reporter assay

HEK293T cells were plated at a density of 5–6 × 10^4^ cells/well in 96-well plates one day before transfection. The cells were co-transfected with 0.2 μg of a firefly luciferase target reporter plasmid, the pMIR-REPORT PirB 3′-UTR plasmid (with either wild-type or mutated miR-181b-binding sites), 0.01 μg of a Renilla luciferase control reporter, and 100 nM miRNA mimics or negative control using Lipofectamine2000 (Invitrogen, California, USA), according to the manufacturer’s instructions. At 6 h post-transfection, the medium in each well was replaced with fresh culture medium. Cells were harvested at 48 h post-transfection and were assayed using a Dual Luciferase System (E1910, Promega). The results are expressed relative to luciferase activity; the values were first normalized to the Renilla luciferase transfection control and then to the firefly/Renilla value of the empty control vector and finally to the corresponding seed mutant reporter control.

### Drug administration

To test whether EA could enhance neurobehavioral functional recovery through regulating miR-181b expression and whether miR-181b could target *pirb* gene silencing after ischemic stroke, the rats received a subcutaneous Alzet osmotic minipump (Alzet, Cupertino, CA, USA)^45^,^46^. The osmotic minipump was implanted beneath the skin via mid-scapular incision. Each pump contained sterile 0.9% saline with miR-181b mimics or miR-181b inhibitors in dimethylsulfoxide (DMSO), which were administered to the animals continuously until the study end (delivering 2 ng/day for 28 days). The miR-181b mimics or miR-181b inhibitors were purchased from GenePharma. After minipump implantation, the rats received EA administration as above method. Rats which had been operated successfully were randomly divided into the following groups: Sham, MCAO, MCAO + EA group, MCAO + EA group+miR-181b inhibitors, MCAO+ miR-181b mimics (n = 6 for each group). After reperfusion, the rats received EA treatment. At 28 d post-MCAO, the mNSS, Rota-rod test and Grip strength were tested. The expression of miR-181b, *pirb* mRNA and PirB protein were also performed at 28 d after reperfusion.

### Neurobehavioral assessments

Neurobehavioral tests were performed by experienced testers who were blind to the experimental groups. Performance on the neurobehavioral tests was assessed at baseline and on different days post-stroke. The following behavioral assessments were used in the present study: ① calculation of the mNSS^47^; ② the rota-rod test^48^; and ③ the grip strengthtest^49^. **The mNSS system**^47^, a composite of motor, sensory, reflex and balance tests, is the most commonly used neurological scoring system in animal studies of focal cerebral ischemia. Neurological deficit was graded on a scale ranging from 0 to 18, with a higher score indicating worse recovery. Baseline performance was determined at 1 day before ischemia. The mNSSs were determined at 28 d after reperfusion. **Rota-rod test.** The rota-rod test was used to evaluate the motor performances of the rats^48^. Three days before MCAO, the rats were trained on an accelerating (4–35 rpm) rota-rod (7750 Ugo Basile, Italy). All animals received a 3-day training program with 3 sessions per day, each lasting for 5 min. On the experimental day, the time spent walking on the rota-rod without falling was measured twice per animal. The interval between each trial was 15 min. The mean time from two trials was calculated for each rat. Baseline performance was determined at 1 day before ischemia. The motor performance was evaluated at 28 days post-MCAO. **Grip strength.** To test the rats’ forelimb grip strengths, an electronic digital force gauge grip strength meter (Columbus Instruments) was used as previously described^49^. Baseline performance was determined at 1 day before ischemia. The forelimb grip strength was evaluated at 28 days post-MCAO. Each rat participated in three successive trials, and the best (highest) score was used for data analysis.

### The effect of miR-181b mimics or miR-181b inhibitors on PirB expression and neurite growth *in vitro*

The OGD model was used as above. The cultures were divided into groups as follows: Normal, OGD, OGD+ miR-181b mimics and OGD+ miR-181b inhibitors group. After rehabilitation of sugar-oxygen, miR-181b mimics or inhibitors were added using Lipofectamine 2000 (Invitrogen) according to the manufacturer’s instructions. At 72 h after OGD, Then the expression of miR-181b, *pirb* mRNA, PirB protein, RhoA, GAP43, β-actin and average length of the longest neurites assay were tested as above.

### Statistical analysis

SPSS 14.0 for Windows (SPSS Inc., Chicago, IL) was used. Statistical analyses were performed with mean ± standard error of the mean (SEM) values using one-way or two-way analysis of variance (ANOVA) with the Bonferroni’s correction. Statistical significance was set at *P* value  < 0.05.

## Additional Information

**How to cite this article**: Deng, B. *et al*. Electroacupuncture enhances rehabilitation through miR-181b targeting PirB after ischemic stroke. *Sci. Rep.* **6**, 38997; doi: 10.1038/srep38997 (2016).

**Publisher's note:** Springer Nature remains neutral with regard to jurisdictional claims in published maps and institutional affiliations.

## Supplementary Material

Supplementary Information
