# Supplementary material for: Electroacupuncture enhances rehabilitation through miR-181b targeting PirB after ischemic stroke
Source: Sci Rep. 2016 Dec 14;6:38997. doi: 10.1038/srep38997 (PMC5155251; doi:10.1038/srep38997)
Supplement: Supplementary Information [file srep38997-s1.pdf]

# Supplementary information

## Electroacupuncture enhances rehabilitation through miR-181b targeting PirB after ischemic stroke

Bin Deng[1,2, *], Fuhai Bai[3, *], Heng Zhou[3], Dandan Zhou[3], Zhi Ma[3], Lize Xiong[3], Qiang Wang[1, 3,†]

**Prof. Michael Nitsche, Editorial Board Member**

Thank you for providing us an opportunity to revise our manuscript. Because our research was an experimental science, so we have to crop the gels/blots in western blots to keep the method convenient and the experiment efficiency costs down. We have mentioned it in the figure legend and full-length gels and blots are included in the supplementary information in the revised file.

Sincerely yours,

Qiang Wang, M.D., Ph.D.

Department of Anesthesiology,
The First Affiliated Hospital of Xi'an Jiaotong University,
Xi'an 710061, Shaanxi Province, China.
E-mail: dr.wangqiang@139.com

**Figure 1-g**

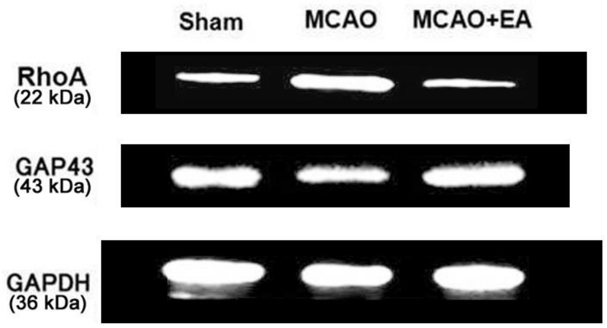


Figure 1. (g) Western blot bands indicating RhoA and GAP43 expression in the ischemic penumbra at 28 d post-MCAO. The full-length gels/blots were cropped for better showing figures in the manuscript.

**Figure 2-a**

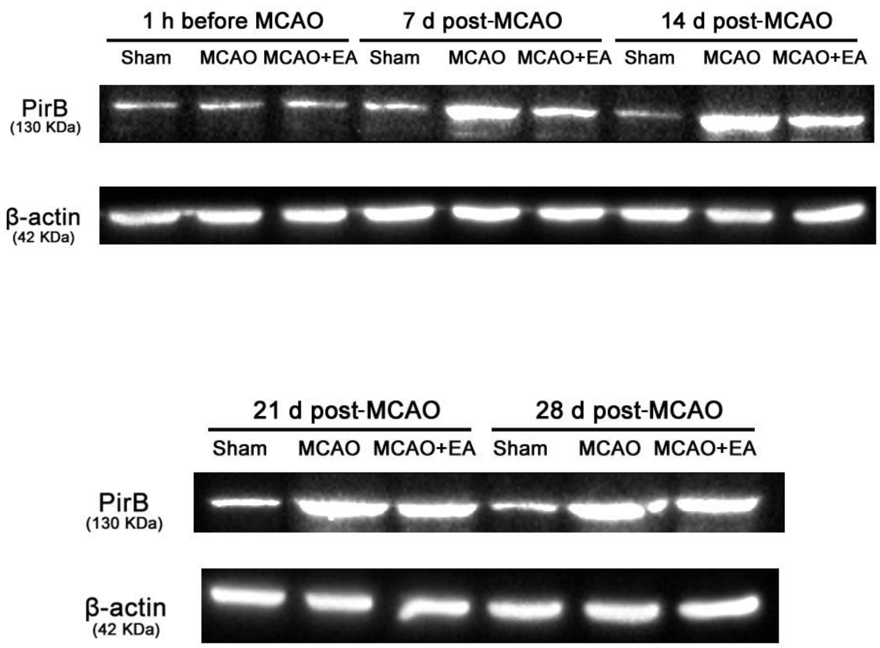


Figure 2. (a) Representative Western blot bands showing PirB expression in the ischemic penumbra. The full-length gels/blots were cropped for better showing figures in the manuscript.

**Figure 3-a/b**

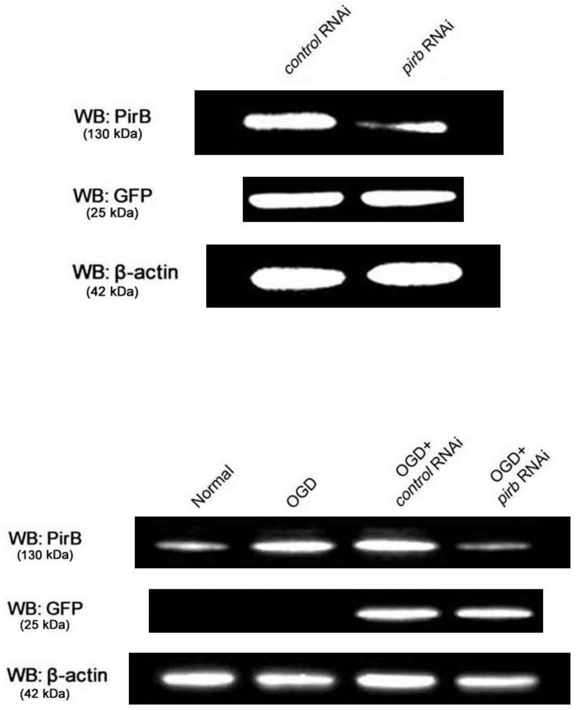


Figure 3. (a) RNAi-mediated silencing of PirB in HEK293 cells. PirB (upper panel), GFP (middle panel) and β-actin (lower panel) expression was detected in extracts by Western blot analysis at 24 h after transfection. (b) RNAi-mediated knockdown of PirB in primary cortical neurons after OGD injury. PirB (upper panel), GFP (middle panel) and β-actin (lower panel) expression was detected in cell extracts via Western blot analysis at 24 h after transfection in each group. The full-length gels/blots were cropped for better showing figures in the manuscript.

**Figure 5-b**

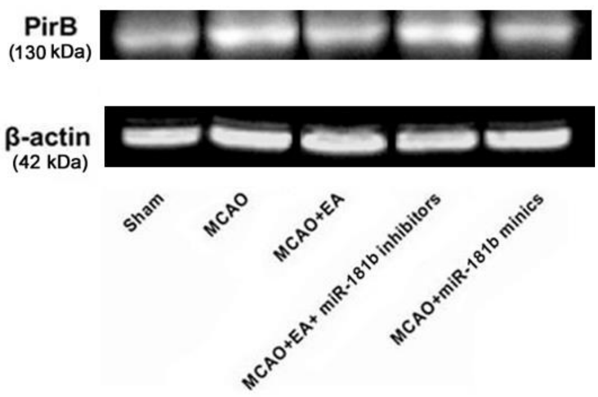


Figure 5. (b) Representative Western blot bands showed PirB expression in the ischemic penumbras at 28 d post-MCAO. The full-length gels/blots were cropped for better showing figures in the manuscript.

**Figure 6-f/h**

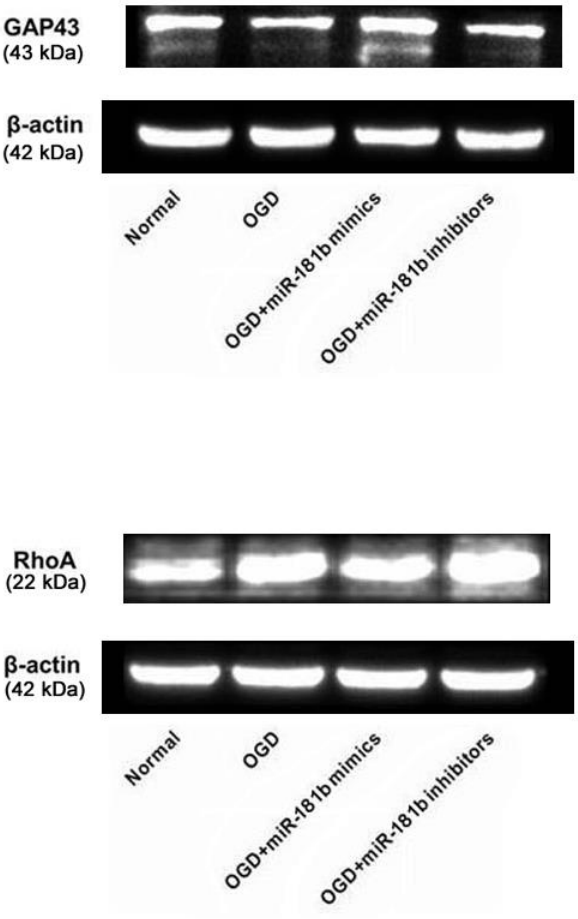


Figure 6. (f, h) Representative Western blot bands showing RhoA and GAP43 expression in neurons at 72 h after OGD injury. The full-length gels/blots were cropped for better showing figures in the manuscript.
